# The association between state mandates of colorectal cancer screening coverage and colorectal cancer screening utilization among US adults aged 50 to 64 years with health insurance

**DOI:** 10.1186/1472-6963-11-19

**Published:** 2011-01-27

**Authors:** Vilma Cokkinides, Priti Bandi, Mona Shah, Katherine Virgo, Elizabeth Ward

**Affiliations:** 1 Surveillance and Health Policy Research, American Cancer Society, Atlanta, USA; 2 Cancer Action Network - American Cancer Society, Washington DC, USA

## Abstract

**Background:**

Several states in the US have passed laws mandating coverage of colorectal cancer (CRC) screening tests by health insurance plans. The impact of these state mandates on the use of colorectal cancer screening has not been evaluated among an age-eligible target population with access to care (i.e., health care insurance coverage).

**Methods:**

We collected information on state mandates implemented by December 31, 2008 and used data on insured adults aged 50 and 64 years from the Behavioral Risk Factor Surveillance System between 2002 and 2008 to classify individual-level exposure to state mandates for at least 1 year. Multivariate logistic regression models (with state- and year- fixed effects, and patient demographic and socioeconomic characteristics) were used to estimate the effect of state mandates on recent endoscopy screening (either flexible sigmoidoscopy or colonoscopy during the past year).

**Results:**

From 1999-2008, twenty-two states in the US, including the District of Columbia passed comprehensive laws requiring health insurance coverage of CRC screening including endoscopy tests. Residence in states with CRC screening coverage mandates in place for at least 1 year was associated with a 1.4 percentage point increase in the probability of utilization of recent endoscopy (i.e., 17.5% screening rates in those with mandates versus 16.1% in those without, Adjusted OR = 1.10, 95% CI: 1.02 - 1.20, p = 0.02).

**Conclusions:**

The findings suggest a positive, albeit small, impact of state mandates on the use of recent CRC screening endoscopy among the target eligible population with health insurance. However, more research is needed to evaluate potential effects of mandates across health insurance types while including controls for other system-level factors (e.g. endoscopy and primary care capacity). National health insurance reform should strive towards a system that expands access to recommended CRC screening tests.

## Background

Colorectal cancer (CRC) is the third leading cause of cancer-related death in the U.S. among both men and women [1]. CRC screening has been shown to reduce colorectal cancer mortality as well as incidence, by virtue of precancerous lesion detection and removal, and it has been recommended for average risk adults aged 50 and older according to clinical guidelines [1-3]. The use of CRC screening has been steadily rising; currently, about half of US adults aged 50 years and older have been screened for CRC with recommended testing modalities (i.e. either a fecal occult blood test or endoscopy tests) [4]. A number of factors are associated with lower utilization of screening including racial or ethnic minority status, lower income and educational level and either total lack of health insurance or inadequate health insurance coverage, particularly with regard to endoscopy for CRC screening [5]. Other barriers to screening include patients' lack of knowledge regarding screening benefits, physician's neglect to reinforce need for screening [5-8]. Based on a survey of health plans conducted in 1999-2000, it was found that most health insurance plans (97%) covered FOBT for average risk (asymptomatic) patients. For the more invasive and expensive CRC screening procedures, such as colonoscopy, a third of the plans covered colonoscopy and nine percent covered flexible sigmoidoscopy but only for patients deemed at high-risk. Also, this survey noted that for eighty-five percent of covering plans, the patient incurred cost-sharing, through co-insurance cost and/or deductibles, for endoscopy testing procedures (average range of charges $130 - $200 for flexible sigmoidoscopy and $400 - $ 650 for colonoscopy) [9]. Patient cost-sharing has been shown to reduce utilization of preventive services and may influence preferences of CRC screening tests [10-12].

In recent years, in an effort to improve CRC screening utilization promulgated by clinical guidelines as well as to expand choice of tests given individual preferences,[13-15] state policy makers have passed legislation in several states to expand coverage for all recommended tests for CRC screening. A few states, however, have implemented policies specifying that either some CRC tests be covered (but not all) or that they be offered as part of the benefits and there remain a large number of states that currently lack regulations [16]. The purpose of this study was to assess the effect of state CRC screening coverage mandates on self-reported utilization of endoscopy (either flexible sigmoidoscopy or colonoscopy testing) among an insured population of US adults aged 50 to 64 years. Endoscopy testing was chosen as the key outcome since it is expected that state mandates requiring coverage of CRC screening would primarily impact access to these expensive CRC screening modalities.

## Methods

### Data source

The Behavioral Risk Factor Surveillance System (BRFSS) is a publicly available state level survey conducted in the United States that measures information on general health and cancer-related risk factors [17]. The BRFSS conducts annual, state-based telephone surveys using a multistage sampling design based on random-digit-dialing methods to select a representative sample of the non-institutionalized adult civilian population, more details about the methodology can be found elsewhere [18]. This study was exempted from IRB review since it used publicly available BRFSS survey data consisting of de-identified respondents records. In this study we used four years of consecutive cross-sectional data from the 2002, 2004, 2006 and 2008 survey years of the BRFSS that collected information on two endoscopy screening modalities (flexible sigmoidoscopy or colonoscopy) [18]. Information about CRC screening mandates was derived from the Health Policy Tracking service of the American Cancer Society Cancer Action Network [16].

### Analytic sample

From the compiled four-year cross-sectional BRFSS survey data, we derived an analytic sample consisting of respondents aged 50 to 64 years who indicated having any health insurance coverage and were residing in 44 states or D.C. (details of state exclusions are explained below).

### Variables

#### CRC legislation

For the purpose of these analyses, states with coverage mandates for CRC screening were those that had implemented comprehensive legislation requiring private insurance plans to cover the full range of colorectal cancer screening tests, including endoscopy procedures, consistent with American Cancer Society guidelines[1] on or before December 31, 2008 [16]. States were excluded if they mandated partial coverage of some tests (Wyoming), "Offers" of coverage (Alabama, Oklahoma and Tennessee), or had voluntary agreements with insurance companies to provide coverage (New York and Vermont) (Table 1). For each respondent, we created a measure of CRC screening legislation 'age' (i.e., time since date of laws' implementation) by calculating the difference in years between the date of passage of the law and the date of interview. A respondent was considered as exposed if they had resided in a state with the law for 1 year or more prior to the date of their interview. Respondents who at the time of interview had mandates for less than 1 year or those in states with no mandates were considered as not exposed. As such respondents in a state in a particular year may have a different exposed/non-exposed status.

**Table 1:** States with Colorectal Cancer Screening Coverage Mandates

State	Date of implementation
Missouri	28-Aug-99
West Virginia	1-Apr-00
Indiana	1-Jul-00
Virginia	1-Jul-00
California	27-Sep-00
Rhode Island	31-Dec-00
Delaware	1-Jan-01
Maryland	1-Jul-01
Texas	1-Sep-01
Connecticut	1-Oct-01
North Carolina	1-Jan-02
Distict of Columbia	13-Apr-02
New Jersey	1-Jul-02
Georgia	1-Jul-02
Nevada	10-Oct-03
Illinois	1-Jan-04
Arkansas	1-Aug-05
Oregon	23-Aug-05
Louisiana	1-Jan-06
Alaska	1-Jan-07
New Mexico	1-Apr-07
Washington	1-Jul-08

Note: States were excluded if they mandated partial coverage of some tests, "Offers" of coverage, or had voluntary agreements with insurance companies to provide coverage

#### CRC screening outcome

In the BRFSS, participants were read this script ("Sigmoidoscopy and colonoscopy are exams in which a tube is inserted in the rectum to view the colon for signs of cancer or other health problems") to enable recall and reporting of endoscopy screening for CRC. Then, participants' information on endoscopy testing was collected by asking these two questions: "Have you ever had either of these exams?" and "How long has it been since you had your last sigmoidoscopy or colonoscopy?" From this information, we defined the outcome as a dichotomous (yes/no) measure of the receipt of an endoscopy CRC screening test in the past year. Though clinical guidelines recommend varying intervals for different types of endoscopy testing for CRC, we used the 'past year' interval in order to minimize potential misclassification of case/control status as a result of respondents' plausible migration between states with and without laws.

#### Covariates

All multivariate analyses controlled for age (categorized as 50 to 54 years old, 55 to 59 years old and 60 to 64 years old), sex (male vs female), race/ethnicity (non-Hispanic White, non-Hispanic Black, Hispanic, and others races), education level (having less than or equal to a high school degree versus having some college or college graduate education), household income level (annual income of less than $25,000, between $25,000 to less than $50,000, over $50,000 and a category of 'missing' was included also since more than five percent of the sample had 'unknown' income information), self-reported health status (categories of fair or poor health, good and better health), and marital status (married, single and divorced/widowed or separated individuals). Even though state mandates primarily affect privately insured populations, the BRFSS did not collect information about the type of health plans among those with health insurance coverage. As we could not restrict our analytic sample to the privately insured group, we considered a state level variable measuring the privately insured proportion among 50-64-year-olds (divided into tertiles and classified as high, medium, and low) for 2002, 2004, 2006 and 2008 as a covariate in all statistical models[19] in addition to controlling for respondents' socioeconomic status.

### Statistical analysis

The independent effect of state CRC screening coverage mandates on CRC screening was evaluated using state- and year- fixed effects models. Multivariate logistic regression analyses were used to calculate adjusted probabilities (predicted marginals) and odds ratios of colorectal cancer screening among respondents with and without state coverage mandates. Models included a state fixed effect that controlled for state factors that do no vary over time (invariant factors) and a year effect that controlled for national secular trends, in addition to individual-level covariates, and state privately insured population rates among those 50-64 years. We present these estimates in comparison with unadjusted probabilities (obtained from unadjusted logistic regression models) in order to understand the influence of other covariates on the relationship between state mandates' exposure and screening probabilities. Further, we performed sensitivity analysis that considered a lag variable in order to capture the effect of mandates at different times after implementation. We tested for significant differences in the adjusted rates among residents in states with mandates for different time periods after implementation (1 year, 2 to 4 years, 5 years or more).

We tested for potential two- and three-way interactions between the mandates and demographic (race and ethnicity) and SES variables (education- and income- level), as it is plausible that the effect of these mandates would be more concentrated among certain groups than others, given that some groups may have had lower access to these tests prior to the passage of these mandates and may thus benefit more after these laws are passed. SAS-callable-SUDAAN Version 10 was used to apply the sampling weights in the calculation of weighted estimates and standard errors (using Taylor Series Linearization Method[20]) that take into account the complex survey design of the BRFSS surveys [18].

## Results

### State mandates and Sample characteristics

Of the 45 states included in the study, 22 states and DC had passed mandates requiring private insurance plans to cover the full range of colorectal cancer screening tests consistent with ACS guidelines on or before December 31, 2008 (Table 1). The analytic sample consisted of 293,626 individuals aged 50-64 years who reported having health insurance coverage (Table 2). Respondents were evenly distributed across years (2002 to 2008) and between the sexes (49% male vs. 51% female). Individuals were mostly in the age-groups of 50-54 (40.6%) and 55-59 years (32.8%), were primarily White, non-Hispanic (79.1%), had college or some college degrees (65.8%), and in higher- and middle-household income groups (≥$50,000: 50.8%, 25,000 to ≤50,000: 23.6%). The majority of respondents reported "better" or "good" health status (81.9%), and were married or members of unmarried couples (74.7%) (Table 2). Across states, the percent of the population 50-64 years who were privately insured averaged over 2002-2008 was generally high and ranged from 69% in Arkansas to 88% in Minnesota (data not shown).

**Table 2:** Descriptive characteristics of US Adults with Health Insurance, aged 50-64 years, surveyed in the period of 2002-2008

Characteristics	Unweighted, N (%)		Weighted %
Year of the survey			
2002	45465	(15.5)	22.3
2004	62271	(21.2)	23.7
2006	82923	(28.2)	26.1
2008	102967	(35.1)	27.9
Age			
50-54	105041	(35.8)	40.6
55-59	100781	(34.3)	32.8
60-64	87804	(29.9)	26.6
Sex			
Male	117328	(40.0)	49.0
Female	176298	(60.0)	51.0
Annual Household income			
<25,000	50398	(17.2)	14.8
25,000-<50,000	75277	(25.6)	23.6
> = 50000	135243	(46.1)	50.8
Missing	32568	(11.1)	10.8
Education			
Less than high school or High School	100382	(34.2)	34.2
Some college or College graduate	192786	(65.8)	65.8
Race			
White, non-Hispanic	246930	(84.4)	79.1
Black, non-Hispanic	19904	(6.8)	8.0
Hispanic	10685	(3.7)	7.4
Other, Multi-racial, non-Hispanic	13615	(4.7)	5.5
Health status			
Excellent or Very good	678834	(51.5)	53.6
Good	396194	(30.1)	30.1
Fair or poor	241939	(18.4)	16.3
Marital status			
Divorced/Widowed/Separated	81354	(27.8)	20.0
Single	21170	(7.2)	5.2
Married/member of unmarried couple	190143	(65.0)	74.7
State privately insured population*			
Low	79047	(26.9)	38.7
Medium	101351	(34.5)	30.2
High	113228	(38.6)	31.1

Note: Missing responses constituted less than 0.8% on all of the above variables, except income for which missing values were coded as a separate category. * State-level variable from the Census[19] and expressed in tertiles of the distribution of the states' proportion of adults aged 50 to 64 years with private insurance.

### Impact of state CRC screening mandates on CRC screening utilization in the past year

In unadjusted analyses, having a state CRC screening mandate for at least 1 year was associated with a 0.4% point increase in the probability of flexible sigmoidoscopy or colonoscopy in the past year (OR: 1.03, 95% CI: 0.99, 1.08, p = 0.09) (Table 3). Adjustment for state- and year-fixed effects, individual covariates, and state-level proportions of privately insured adults resulted in a stronger mandate effect compared to unadjusted analyses; the probability of CRC screening was 1.4% points higher among individuals residing in states with mandates than those without (17.5% vs. 16.1%, OR: 1.10, 95% CI: 1.02, 1.20, p = 0.02) (Table 3). Additionally, we considered the possibility that state mandates require more than one year of implementation to be effective in changing CRC utilization patterns. CRC utilization rates did not differ significantly between respondents in states with laws in place for different time intervals.

**Table 3:** Impact of Colorectal Cancer Screening coverage mandates on Endoscopy Screening ^a ^Use in Past Year among US Adults with Health Insurance aged 50-64 years, 2002-2008

Characteristic	Unadjusted prevalence (95% CI)	OR (95% CI)	**Adjusted prevalence **^**b **^**(95% CI)**	OR (95% CI)
CRC Screening Mandate				
State mandate for at least 1 year	16.9 (±0.4)	1.03 (0.99, 1.08)	17.5 (±0.7)	1.10 (1.02, 1.20)*
State mandate for less than 1 year or No state mandate	16.5 (±0.4)	1.00	16.1 (±0.5)	1.00
Covariates				
Year of the survey				
2008	17.9 (±0.4)	1.23 (1.16, 1.3)***	17.5 (±0.5)	1.16 (1.09, 1.24)***
2006	17.4 (±0.6)	1.18 (1.12, 1.25)	17.2 (±0.5)	1.13 (1.06, 1.21)
2004	16.0 (±0.6)	1.07 (1.01, 1.14)	16.1 (±0.6)	1.05 (0.98, 1.12)
2002	15.1 (±0.6)	1.00	15.5 (±0.6)	1.00
Age				
50-54	15.2 (±0.4)	0.77 (0.73, 0.81)***	15.1 (±0.4)	0.76 (0.72, 0.79)***
55-59	16.8 (±0.4)	0.87 (0.83, 0.91)	16.7 (±0.5)	0.86 (0.81, 0.90)
60-64	18.9 (±0.6)	1.00	19.0 (±0.5)	1.00
Sex				
Male	16.9 (±0.4)	1.04 (1.00, 1.08)*	16.8 (±0.4)	1.01 (0.97, 1.05)
Female	16.4 (±0.4)	1.00	16.6 (±0.3)	1.00
Annual Household Income				
<25,000	15.5 (±0.6)	0.86 (0.81, 0.91)***	14.7 (±0.7)	0.80 (0.75, 0.86)***
25,000-<50,000	15.4 (±0.6)	0.85 (0.81, 0.89)	15.5 (±0.5)	0.85 (0.81, 0.89)
> = 50000	17.5 (±0.4)	1.00	17.7 (±0.4)	1.00
Missing	17.2 (±0.8)	0.98 (0.92, 1.04)	17.1 (±0.8)	0.96 (0.89, 1.02)
Education				
Less than high school or High School	15.4 (±0.4)	0.87 (0.83, 0.90)***	15.3 (±0.5)	0.86 (0.82, 0.90)***
Some college or College graduate	17.4 (±0.4)	1.00	17.4 (±0.4)	1.00
Race				
Black, non-Hispanic	20.7 (±1.2)	1.33 (1.24, 1.43)***	21.1 (±1.2)	1.38 (1.28, 1.48)***
Hispanic	15.3 (±1.4)	0.92 (0.82, 1.03)	16.3 (±1.5)	1.01 (0.90, 1.13)
Other, Multi-racial, non-Hispanic	16.5 (±1.6)	1.00 (0.89, 1.14)	16.9 (±1.7)	1.05 (0.93, 1.19)
White, non-Hispanic	16.4 (±0.2)	1.00	16.3 (±0.3)	1.00
Health status				
Fair or poor health	19.2 (±0.8)	1.24 (1.18, 1.3)***	20.4 (±0.8)	1.36 (1.29, 1.44)***
Good or better health	16.1 (±0.2)	1.00	15.9 (±0.3)	1.00
Marital status				
Divorced/Widowed/Separated	15.9 (±0.6)	0.92 (0.88, 0.96)***	15.9 (±0.5)	0.92 (0.88, 0.97)***
Single	15.2 (±1.2)	0.87 (0.8, 0.95)	15.2 (±1.1)	0.87 (0.80, 0.96)
Married/member of unmarried couple	17.0 (±0.4)	1.00	17.0 (±0.3)	1.00
State privately insured population				
Low	16.4 (±0.5)	0.92 (0.88, 0.96)***	16.9 (±1.1)	1.02 (0.89, 1.17)
Medium	16.2 (±0.4)	0.91 (0.87, 0.94)	16.6 (±0.7)	1.00 (0.93, 1.07)
High	17.6 (±0.4)	1.00	16.6 (±0.9)	1.00

* p < 0.05, ** P < 0.01, ***p < 0.001; 95% CI: 95% confidence interval; OR: Odds ratio; CRC: Colorectal Cancer

^**a **^Endoscopy screening is defined as having ever had either sigmoidoscopy and colonoscopy and reporting receipt of any of these screening tests in the past year. ^**b **^Adjusted prevalences are predicted marginals derived from multivariate logistic models predicting endoscopy use, that adjusted for covariates listed above, in addition to state fixed effects.

The two-way interaction terms between state mandates and demographic- and socioeconomic- variables were not statistically significant; indicating that the effect of mandates did not differ significantly between subgroups of respondents based on their race/ethnicity, education, and household income levels (data not shown). However, linear contrasts of the adjusted probabilities indicated that the effect of mandates was significant and stronger in higher-educated respondents (16.7% vs. 18.3%, contrast: 1.6% points, p = 0.01) but not in lower-educated respondents (15% vs. 15.8%, contrast: 0.8%, p = 0.12). A three-way interaction variable between state mandates, a collapsed two-level version of the household income (low- and middle-income: < $50,000, and high income group: ≥$50,000), and education level (lower-education: less than high school or high school graduate, and higher-education: college graduate or some college graduate) was statistically significant (p < 0.005). Linear contrasts of the adjusted probabilities from the interaction term indicated that among low- and middle-income individuals, the effect of state mandates was positive and significant only among higher-educated individuals (14.4% vs. 17.9%, contrast: 3.5% points, p < 0.001) but not among lower-educated individuals (13.7% vs. 14.1%, contrast: 0.4% points, p = 0.5). Conversely, state mandates resulted in marginally higher CRC utilization rates among lower educated-high income individuals (15.3% vs. 18.0%, contrast: 2.7% points, p < 0.05), but not among high educated-high income individuals (17.9% vs. 19.1%, contrast: 1.2% points, p = 0.12).

## Discussion

In this study, we provide the first assessment of the utilization effects of state mandates requiring private insurers to cover recommended CRC screening. The main findings suggest that state mandates are positively associated with utilization of CRC endoscopic screening (1.4% absolute increase in probability of past-year endoscopy test use) in adults aged 50 to 64 years with access to care. In addition, our results suggest that among low- and middle- income groups, mandates seemed to have benefited higher-educated groups more than lower-educated, whereas education-related disparities in high income groups appear to have been reduced as a result of mandates. Even though we know of no prior study that has examined this association in a non-Medicare insured population of adults aged 50 to 64 years, our study findings are generally consistent with previous studies conducted among the elderly Medicare beneficiaries, showing positive effects of policy changes [21-24].

In reflecting on these study's finding suggesting a small effect of state CRC screening mandates (in absolute percentage differences), we have to consider several contextual issues and challenges that may stimulate additional health policy research endeavors. First, there is the issue that state-mandated benefits for CRC screening are limited in their reach because of certain exemptions. State mandates only apply to certain types of insurance coverage in the commercial private marketplace. It is the case that, the federal Employee Retirement Income Security Act (ERISA) exempts states from regulating self-funded employer-based health plans from state laws that govern health insurance [25,26]. We were unable to distinguish and exclude individuals subject to these self-insured plans, potentially biasing our estimates towards the null. Secondly, we had very limited information on respondents' health insurance plans. We were unable to restrict our sample to privately insured respondents, although we considered the inclusion of a state measure of privately insured populations as an adjustment factor. Further, we could not account for potential differences across types of private insurance (e.g. managed-care plans, fee-for-service, high-deductible health service accounts, etc); such variations in types of private insurance arrangements may play a role in utilization of medical services [27]. Thirdly, although mandated benefits for CRC screening may have expanded options for CRC testing modalities they do not address the cost-sharing requirements for the more expensive modalities (i.e., sigmoidoscopy and colonoscopy). Studies have shown that patient-cost sharing requirements influence preferences for specific CRC screening tests and are likely to reduce utilization of preventive services [10-12]. Another issue is that despite state-mandated benefits resulting in changes to health plan policies for CRC screening, certain socioeconomic segments may still access these expanded benefits at disproportionately lower/higher rates vis-à-vis other groups. Our results suggest CRC endoscopy uptake patterns after passage of state mandates may be a function both individuals' education and income levels which may in turn relate to other influences such as patients' knowledge of expanded benefits and eligibility requirements and receipt of healthcare provider CRC screening recommendation [28-30]. Despite state mandates, patients' ability to obtain an endoscopy test (or a referral) may be affected by the availability of endoscopy-infrastructure services/providers. Thus, future studies are needed to better characterize whether and how these issues/concerns affect the robustness/validity of our findings. Lastly, our study was limited by its reliance on self-reported survey data and so future studies based on claims and/or clinic records are needed.

## Conclusion

Screening for colorectal cancer is a beneficial, cost-effective way to advance the public's health by reducing the incidence and mortality of CRC [1,5]. Colorectal cancer screening has been designated as one of the highest priority preventive services by the National Commission on Preventive Priorities based on the burden of disease that could be prevented [31,32]. It is increasingly recognized that all age-eligible asymptomatic adults have adequate and affordable access to all recommended screening procedures in order to improve survival and quality of life [27]. Our study suggests that state mandates for CRC screening test coverage may increase utilization among insured individuals. Through advocacy efforts, colorectal cancer screening and prevention has been part of the national healthcare reform discussions and much is expected with the recent passage of landmark federal legislation, the Patient Protection and Affordable Care Act [33]. This Act requires all new private health plans cover colorectal cancer screening tests with a U.S. Preventive Services Task Force (USPSTF) rating of "A" or "B" without any out-of-pocket costs to patients [34]. Now, with the advent of its implementation, there is yet the potential for future improvements in CRC screening test utilization as it intends to prioritize access to preventive care for all, including previously uninsured individuals.

## Competing interests

The authors declare that they have no competing interests.

## Authors' contributions

VC initiated the research idea, designed the study, and wrote the manuscript. PB contributed to the study design, conducted the analysis and wrote parts of the manuscript. MS provided valuable support in the compilation of state mandates information as well as review/editing of the manuscript. KV provided valuable comments on the analysis and editorial suggestions on the manuscript. EW provided valuable comments and editorial suggestion on the manuscript. All authors read and approved the final manuscript.

## Pre-publication history

The pre-publication history for this paper can be accessed here:

http://www.biomedcentral.com/1472-6963/11/19/prepub
